# Characterization of an aged alkali-activated slag roof tile after 30 years of exposure to Northern Scandinavian weather

**DOI:** 10.1039/d2ra04456k

**Published:** 2022-09-12

**Authors:** Tero Luukkonen, Juho Yliniemi, Brant Walkley, Daniel Geddes, Ben Griffith, John V. Hanna, John L. Provis, Paivo Kinnunen, Mirja Illikainen

**Affiliations:** Fibre and Particle Engineering Research Unit, University of Oulu P.O. Box 8000 FI-90014 Oulu Finland juho.yliniemi@oulu.fi; Department of Chemical and Biological Engineering, The University of Sheffield Sheffield S1 3JD UK; Department of Materials Science and Engineering, The University of Sheffield Sheffield S1 3JD UK; Department of Physics, The University of Warwick Coventry CV4 7AL UK

## Abstract

Alkali-activated materials (AAMs) have been known as an alternative cementitious binder in construction for more than 120 years. Several buildings utilizing AAMs were realized in Europe in the 1950s–1980s. During the last 30 years, the interest towards AAMs has been reinvigorated due to the potentially lower CO_2_ footprint in comparison to Portland cement. However, one often-raised issue with AAMs is the lack of long-term studies concerning durability in realistic conditions. In the present study, we examined a roof tile, which was prepared from alkali-activated blast furnace slag mortar and exposed to harsh Northern Scandinavian weather conditions in Turku, Finland, for approximately 30 years. Characterization of this roof tile provides unique and crucial information about the changes occurring during AAM lifetime. The results obtained with a suite of analytical techniques indicate that the roof tile had maintained excellent durability properties with little sign of structural disintegration in real-life living lab conditions, and thus provide in part assurance that AAM-based binders can be safely adopted in harsh climates. The phase assemblage and nanostructural characterization results reported here further elucidate the long-term changes occurring in AAMs and provide reference points for accelerated durability tests and thermodynamic modelling.

## Abstract 2

The phase assemblage and nanostructural characterization results reported here further elucidate the long-term changes occurring in alkali activated blast furnace slag binders.

## Graphical abstract

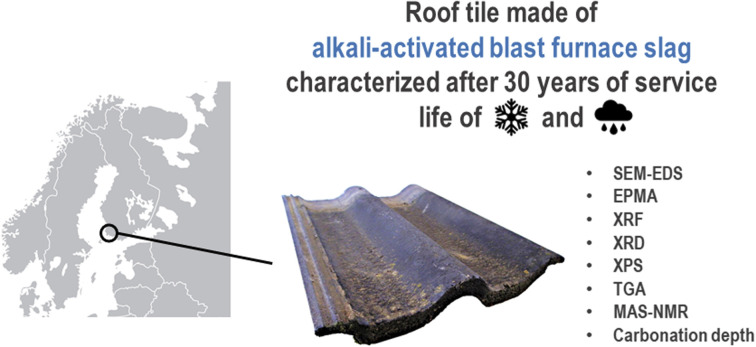

## Introduction

The cement industry plays an important role in limiting global warming: CO_2_ released from the calcination of limestone and generation of the required over 1000 °C temperature constitute approximately 8% of all anthropogenic CO_2_ emissions.^1^ Several carbon footprint mitigation strategies for the cement industry have been proposed: alternative fuels or raw materials; replacement of Portland cement partially by supplementary cementitious materials; or using alternative binders not containing Portland cement at all.^2^ Alkali-activated materials (AAMs), and geopolymers as their sub-group, have been actively studied as one alternative binder.^3^ The increased interest towards AAMs during the last 30 years is due to the fact that their global warming potential can be up to 96% lower than with Portland cement.^4^ This is, however, highly dependent on the mix design, local availability of materials, requirement for heat curing, and so on.^4–7^ Moreover, AAMs can be designed to exhibit better material performance, such as higher mechanical strength and better durability, in comparison to Portland cement-based concrete.^8^

The differences between AAMs and Portland cement-based concrete can be understood by their chemical composition and microstructure. The main phase of Portland cement binder is calcium–silicate–hydrate gel (C–S–H), which consists of Si atoms primarily in dimeric (Q^1^) and ‘chain-like’ (Q^2^) environments.^9^ AAMs, on the other hand, contain aluminium-substituted C–S–H (*i.e.*, C–A–S–H) or sodium–aluminum–silicate–hydrate (N–A–S–H) gels when using high and low-calcium raw materials, respectively.^10^ Also intermediate structures and secondary products such as Mg–Al-layered double hydroxides (LDHs), AFm group calcium aluminates, and zeolites can be formed.^10^ The molecular level structure of the C–A–S–H gel resembles tobermorite^11^ with partially cross-linked chains containing Q^2^ and Q^2^(1Al) environments.^9^ The N–A–S–H gel, on the other hand, consist of three-dimensional aluminosilicate network with Q^4^(2Al) and Q^4^(3Al) centers.^9^

The concept of alkali activation has been known for over 120 years: one of the earliest references dates back to 1895.^12^ In the 1950s–1980s, AAMs were applied for various construction projects in Europe: some of the buildings are still in use, for instance in Belgium and Ukraine.^13,14^ In the former Soviet Union, the AAMs were completely by-product-based, as ground granulated blast furnace slag (BFS) was used as the precursor and the activators were based on by-products from alumina and nitrogen production.^15^ The drivers for using AAMs in this time period were a comparatively low price^13,16^ and shortage of Portland cement.^17^ In the 1970–1980s, so called F-concrete was developed in Finland: its binder was based on BFS activated by sodium hydroxide and silicate solution containing lignosulfonate as superplasticizer.^18^ Even though the material was able to meet the technical requirements for precast concrete elements manufacturing,^18^ it did not eventually reach wide-scale use. In recent years, Australia has been one of the forerunners in the commercial use of AAMs. As examples, the University of Queensland's Global Change Institute and the Toowoomba Wellcamp Airport construction projects in 2013 and 2014, respectively, used alkali-activated concrete.^19,20^ In these recent projects, AAMs have been used mainly because of their lower carbon footprint, which will be more and more closely correlated to the costs in the future due to increased use of carbon taxes, for instance.^21^

As described above, there is a track record of over 70 years of using AAMs in full-scale construction. Nevertheless, the long-term behavior and durability of AAMs employed in these past projects has been scarcely documented in the scientific literature. Buchwald *et al.*^13^ studied concrete specimens based on so-called Purdocement (BFS activated by Na_2_CO_3_ and Ca(OH)_2_), which were sampled from still-existing buildings in Brussels, Belgium, constructed in the 1950s. They observed that the material had been vulnerable to carbonation, but the compressive strength had remained high (≈50 MPa).^13^ Xu *et al.*^15^ characterized alkali-activated concrete samples from Kyiv, Ukraine, prepared between 1964–1982 in which BFS was used as a precursor and alkali carbonate or carbonate/hydroxide as an activator. The quality of the concrete had remained good and compressive strength increased (by 30–247%) in comparison to the initial design strength even though the material was exposed to repeated freeze–thaw cycles for several decades.^15^ This was explained by a cyclic process in the strength development in which Ca and Si dissolve from BFS, followed by CaCO_3_ formation and Si concentration increase until C–S–H precipitates and takes Ca from CaCO_3_.^15^ Then, the released CO_3_^2−^/HCO_3_^−^ continues to react with remaining BFS particles.^15^ Mancini *et al.*^22^ investigated blended Portland cement and BFS concretes, which had been exposed to river or sea water for up to seven years. They observed that the prevalent conditions have major impact on the iron oxidation reactions. Pasupathy *et al.*^23^ studied BFS-based alkali-activated concrete exposed to marine conditions in the splash zone for four years: their findings showed higher tendency for scaling and chloride diffusion, and lower chloride binding capacity in comparison to Portland cement–concrete. These above-listed studies show that the long-term properties in real-life conditions are strongly dependent on mix design and environmental factors. Thus, more case studies are needed.

In the present paper, we report the phase assemblage of alkali-activated BFS mortar prepared in 1988 in Finland. The studied material was not, however, based on the F-concrete, which was being developed in Finland at the same time. The studied specimen is a roof tile, which had been in use, and thus exposed to harsh weather conditions of the Northern Scandinavia (in Turku, Finland) for more than 30 years. The Köppen–Geiger classification of the sampling region belongs to the warm-summer humid continental climate^24^ with six or seven months of annual exposure to freeze–thaw weathering (*i.e.*, temperature fluctuating frequently above and below 0 °C). The mean temperature during winter time is −5.3 °C,^25^ while the lowest recorded temperature has been −34.8 °C.^26^ The average number of precipitation days (raining or snowing) in a year has varied between 16–21 days,^26^ and the yearly average precipitation sum in that region is around 630 mm.^25^ To gain understanding about the durability and other changes, the specimen was characterized thoroughly for chemical and mineralogical composition, micro- and nanostructure, and carbonation depth.

## Materials and methods

### Mix design and preparation of the roof tile

The examined roof tile was a commercial product prepared in 1988 by Partek Oyj Abp in Parainen, Finland. The binder in the roof tile contained BFS, Ca(OH)_2_, and sodium silicate. The manufacturing was conducted by co-grinding a dry mixture of 96 wt% BFS and 4 wt% of Ca(OH)_2_. The mortar was then prepared by blending the aforementioned dry mixture and sodium silicate solution (molar SiO_2_/Na_2_O = 1) in a weight ratio of 94 to 6, respectively, and adding 330 kg m^−3^ of sand. The role of Ca(OH)_2_ was to act as a setting accelerator. The ground-granulated BFS was from Rautaruukki steel mill (Raahe, Finland) and its composition was 35.76 wt% of SiO_2_, 39.44 wt% of CaO, 8.31 wt% of Al_2_O_3_, and 9.30 wt% of MgO as analyzed from the unreacted slag particles remaining in the binder with a scanning electron microscope with an energy dispersive spectroscope (SEM-EDS, see details below). The Na_2_O content in the mix design was 3–4 wt% of the amount of BFS. The binder content was 280–460 kg m^−3^. The roof tiles were prepared with an extrusion pressure process.

Regarding the binder content and activator modulus, the mix design of roof tile is closely similar to mix designs “S3a” and “S1b” used in the RILEM AAM round robin tests.^27,28^ However, as the roof tiles were prepared with an extrusion pressure process, the water-to-binder ratio is lower. Furthermore, BFS used here had higher MgO (9.3 wt% *vs.* 6.4 wt%) content and lower CaO (39.4 wt% *vs.* 41.4 wt%) and Al_2_O_3_ (8.3 wt% *vs.* 11.3 wt%) contents compared to the BFS used in the RILEM round robin tests. The overall composition of the roof tile based on X-ray fluorescence (XRF) analysis is presented in Table 1. The XRF analysis also contains contribution from sand, which increases the total SiO_2_ and Al_2_O_3_ contents and decreases the contents of other oxides.

**Table 1:** XRF analysis of the roof tile sample

Oxide	[Weight%]
SiO_2_	55.95
Al_2_O_3_	10.5
CaO	9.32
Na_2_O	3.82
Fe_2_O_3_	2.57
MgO	2.35
K_2_O	2.07
SO_3_	0.81
TiO_2_	0.42
MnO	0.17
P_2_O_5_	0.08
Cr_2_O_3_	0.05
Cl	0.04
ZrO_2_	0.02
Loss-on-ignition (950 °C)	6.00
Sum	94.17

### Sampling

**Fig. 1:**
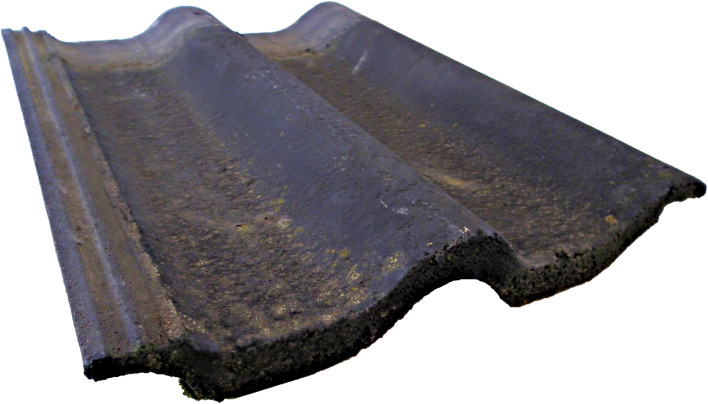
Appearance of the roof tile specimen (size approximately 430 mm × 330 mm × 25 mm) after sampling: no signs of disintegration or damage. The sample piece (∼30 mm × 100 mm) for characterization was cut from the bottom left-hand side corner of the tile.

The roof tile was installed in 1988 to a house located in Turku, Finland. The tile remained in use until May 2019 (approximately 31 years) after which it was removed for examination (Fig. 1). The dimensions of the tile were approximately 430 mm × 330 mm × 25 mm. A piece of the tile was cut for analysis and, when needed, pieces were crushed manually to particle sizes below 2 mm. While there is no quantifiable data about the compressive strength of the sample, the effort required to saw off a piece of the tile with a diamond tip blade demonstrated remarkable robustness of the material.

### Characterization

SEM-EDS (Zeiss Ultra Plus) was used for semi-quantitative microanalysis of the binder matrix in the mortar. Analyses were conducted using a backscatter electron detector with 15 kV acceleration voltage and 8.5 mm working distance. To analyze the binder gel, point analysis were manually selected on areas that did not contain unreacted BFS particles or aggregate. The samples were prepared by impregnating tile pieces in epoxy resin (Buehler) and polishing with P120, P240, and P1200 abrasive grinding paper and ethanol flushing to reveal cross-sections. The samples were carbon-coated before the analysis.

The elemental maps were composed by using a field emission electron probe microanalyzer (EPMA) JEOL JXA-8530FPlus.

X-ray diffraction (XRD) was performed to identify and quantify crystalline phases with a Rigaku Smartlab diffractometer (9 kW Cu X-ray source) in the range of 5–120 °2*θ* using 6 °2*θ* per min scan speed.

Thermogravimetric analysis (TGA) of crushed roof tile pieces were conducted by heating from 22 to 1000 °C at 5 °C min^−1^ in a N atmosphere to detect the changes in mass. Differential thermogravimetric analysis (DTG) was conducted by differentiating the TG curve with the Origin 2018b software (OriginLab Corporation). For TGA, samples were crushed and sand particles were separated by sieving.

Solid state single pulse ^29^Si, ^27^Al, and ^23^Na magic angle spinning (MAS) NMR data were obtained to examine the local structure of the reaction products in each sample. All ^29^Si and ^23^Na spectra were acquired at 11.7 T on a Bruker Avance III HD 500 spectrometer operating at Larmor frequencies 99.35 MHz and 132.29 MHz, respectively. A Bruker 4.0 mm dual resonance CP/MAS probe was used enabling a MAS frequency of 12.5 kHz. ^29^Si MAS NMR spectra were acquired using a 5.5 μs π/2 excitation pulse, a measured 90 s relaxation delay, a total of 256 FIDs per spectrum. ^1^H–^29^Si cross-polarization (CP) MAS NMR experiments were performed using a ^29^Si π/2 pulse width of 1.7 μs, an initial ^1^H non-selective (π/2) pulse width of 2.5 μs, a recycle delay of 1.5 s and a contact pulse of 1.7 ms. A nominal ^1^H decoupling field strength of 80 kHz was employed during acquisition and 10 240 transients were collected per experiment. ^23^Na MAS NMR spectra were acquired using a 3 μs non-selective (π/2) excitation pulse, a measured 10 s relaxation delay and a total of 128 scans per spectrum. All ^29^Si and ^23^Na spectra were referenced to the IUPAC primary references, pure tetramethylsilane (TMS) (*δ*_iso_ = 0 ppm) and 1.0 M aqueous NaCl_(aq)_ (*δ*_iso_ = 0 ppm), respectively.^29^

Single pulse ^27^Al MAS data were acquired at 11.7 and 20.0 T using Bruker Avance III HD 500 and 850 spectrometers operating at the Larmor frequencies 130.28 and 221.49 MHz. A Bruker 4.0 mm dual resonance CP/MAS probe was used enabling a MAS frequency of 12.5 kHz. ^27^Al MAS NMR data were acquired at 11.7 T using a 1.7 μs π/2 excitation pulse, a measured 10 s relaxation delay, a total of 64 FIDs per spectrum. A 3.2 mm HXY probe was used which enabled a spinning frequency of 20 kHz. Pulse calibration and chemical shift referencing were carried out using the IUPAC primary reference of 1.1 M solution Al(NO_3_)_3_ (*δ*_iso_ = 0 ppm)^29^. ^27^Al MAS NMR data were acquired at 20.0 T using a ‘non-selective’ 18 μs π/2 pulse was measured allowing for a ‘selective’ 1 μs π/12 to be implemented. A minimum of 2600 scans was acquired per spectrum, with a recycle delay of 1 s between subsequent acquisitions. 2D ^27^Al 3QMAS z-filter experiments were attained at 20.0 T (*ν*_0_ = 221.49 MHz) using a Bruker Avance Neo spectrometers. A 4 pulse z-filter 3QMAS pulse sequence (excitation–conversion – π/2 − π/2–acquire) was implemented where a calibrated 6.2 μs ‘hard’ excitation pulse, 1.8 μs conversion pulse and 11 μs ‘soft’ z-filter pulses. A total of 64 slices were acquired per spectrum and 96 scans were obtained per slice.

X-ray photoelectron spectroscopy (XPS) was used for the determination of the oxidation state of S and Fe. A powdered sample of the inner-section of the roof tile was measured. The measurements were carried out using a Thermo Fisher Scientific ESCALAB 250Xi XPS System. C 1s (284.8 ev) was used as a standard reference and background is subtracted in the spectra.

Carbonation of the mortar was examined with the phenolphthalein method.^30^ A freshly revealed cross-section of a cut piece was swept with paper to remove dust and a phenolphthalein solution (1 g of phenolphthalein [VWR Chemicals] dissolved in 100 mL of water–ethanol solution) was sprayed on the surface to reveal the carbonation depth. Pink color formation indicates that pH is >8 (*i.e.*, no carbonation) and no color that pH is less than 8.

## Results and discussion

### Microstructure and composition

**Fig. 2:**
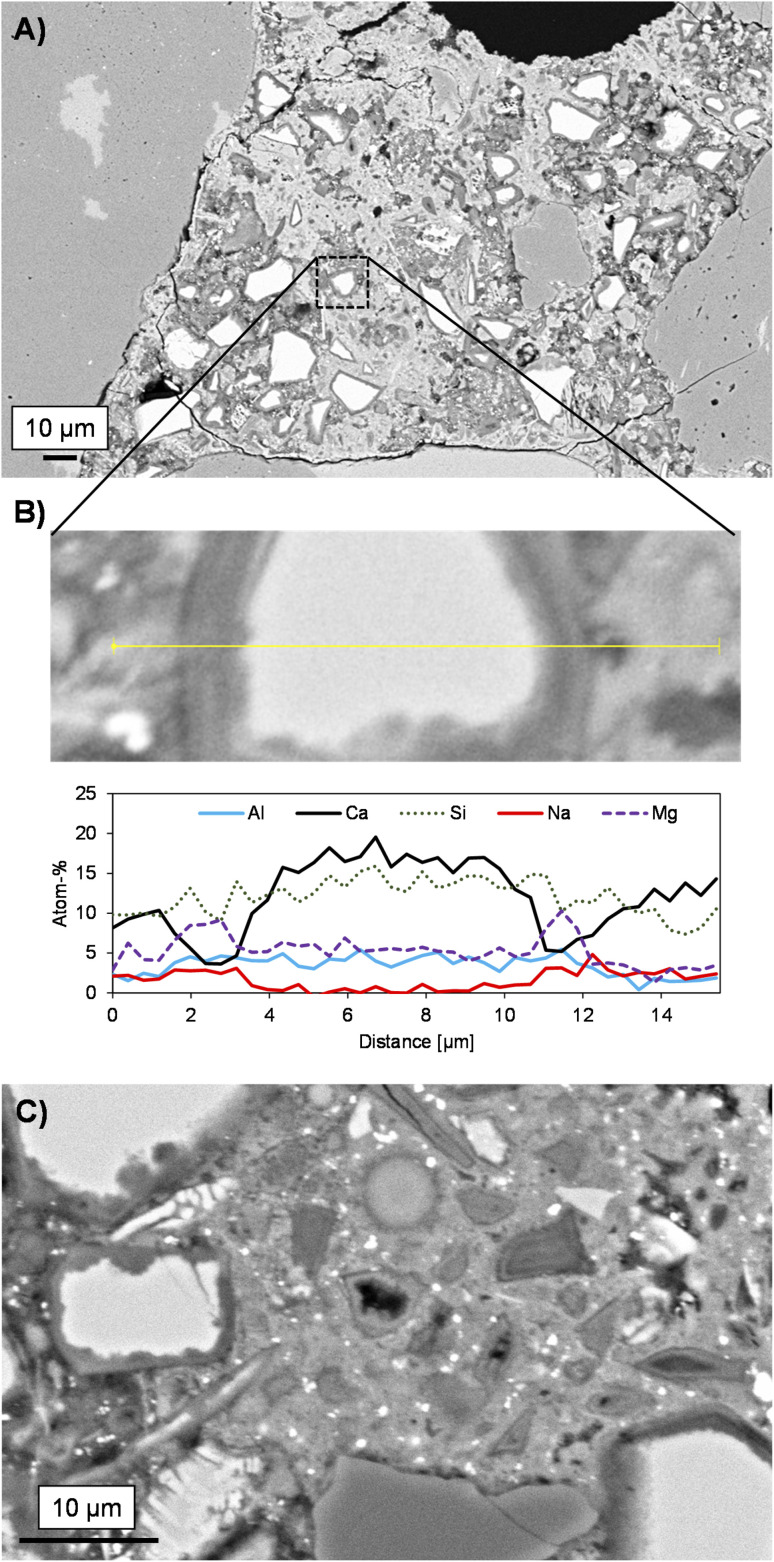
Microstructure of sample cross-sections at different magnifications (A and C) and a line-scan analysis over a partially reacted blast furnace slag particle (B) as determined with SEM-EDS.

The microstructure of the roof tile resembles typical alkali-activated BFS mortar with unreacted BFS particles acting as microfillers (Fig. 2A). The cracks in Fig. 2 are likely caused by the sample polishing and pre-treatment for SEM-EDS analysis. No sign of aggregate alkali–silica reaction (ASR) is observed. This gives further confidence regarding the ASR test methods for the assessment of AAM durability described in^27^ in which it was concluded that AAMs does not possess similar risk for ASR as Portland cement despite the high alkali content in the mix design. The interfacial transition zone can be seen as an occasional, up to ∼10 μm thick, higher porosity area around the aggregate particles but there is not an evident mineralogical distinction from the bulk binder regions that can be detected in these images.

**Fig. 3:**
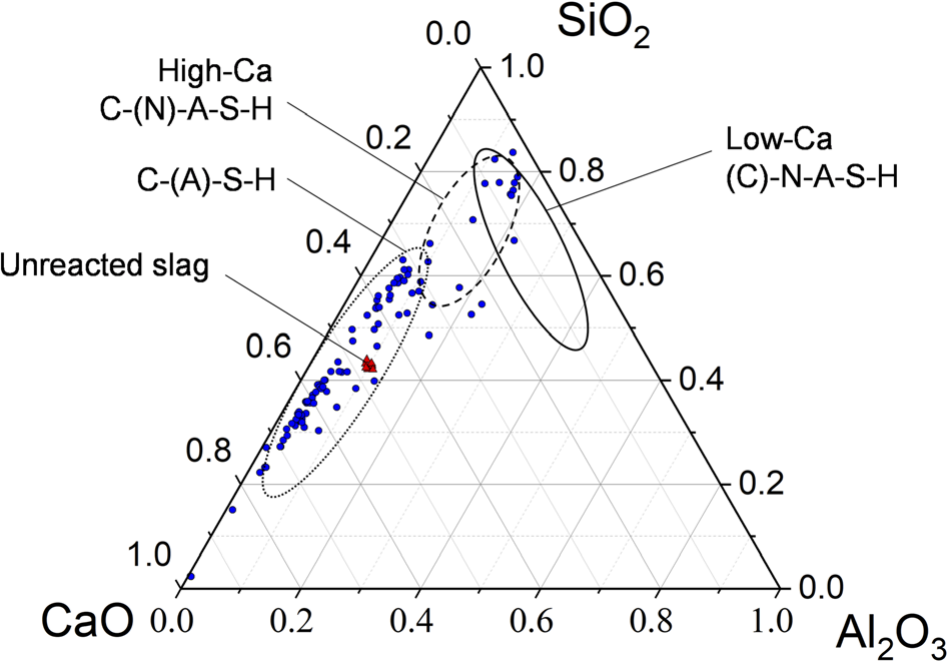
Pseudo-ternary diagram showing the chemical composition (as atomic%, renormalized to CaO–Al_2_O_3_–SiO_2_ only) of the binder matrix (blue circles) and unreacted blast furnace slag (red triangles) as determined by SEM-EDX microanalysis.

**Fig. 4:**
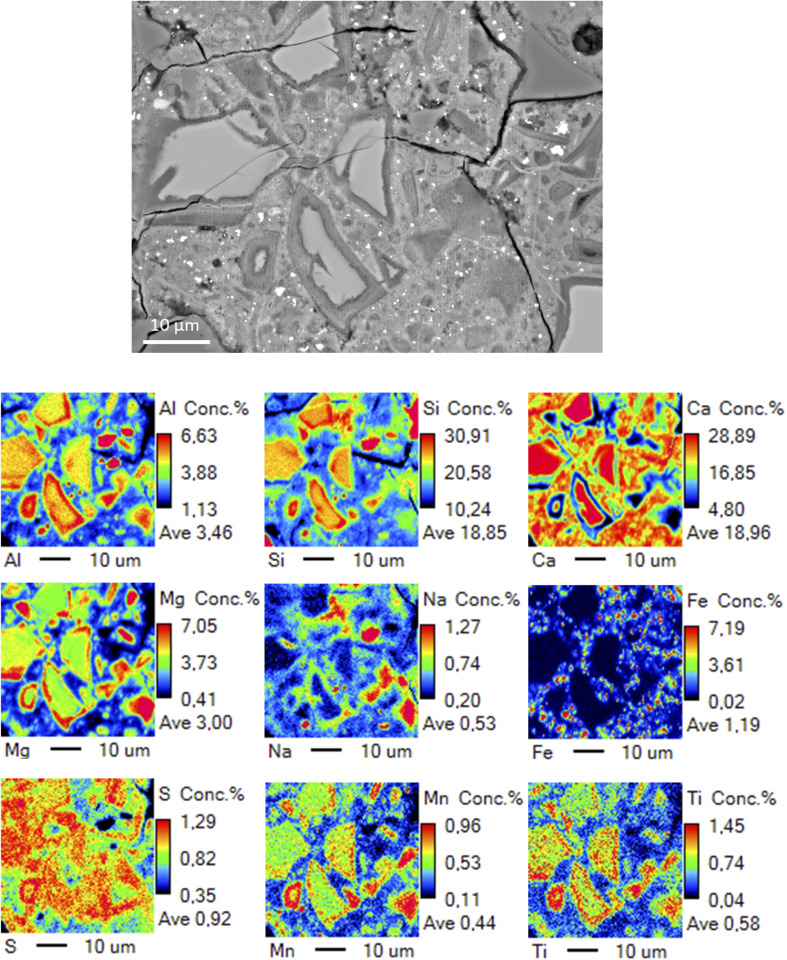
Elemental maps of a sample cross-section as determined by EPMA.

The binder matrix appears to consist of two distinct phases as indicated by the lighter and darker colors in Fig. 2A. This is likely related to the calcium content variation as summarized in the ternary diagram (Fig. 3). The hydrated rims around slag particles are ∼2 μm in width. The line-scan analysis over a BFS particle (Fig. 2A) and the elemental map (Fig. 4) both indicate that the Mg and Al contents are higher in the rims, *i.e.*, interface zone (∼2 μm thick) between the binder gel and the BFS particle: this is likely caused by the low mobility of Mg dissolved from BFS in the high pH conditions and the subsequent precipitation as Mg(OH)_2_ and Mg–Al LDHs, which are often detected in alkali-activated BFS cements.^31,32^ Mg/Al ratio in this region is 2.0, which is consistent with the chemical composition of quintinite group LDH.^33^ It is also noted that Fe is not present in this region, *i.e.*, Fe^2+^ and Fe^3+^ have not replaced Mg^2+^ and Al^3+^, respectively, in the LDH structure over time. In contrast, Mn is enriched in these hydrated regions with an amount of up to 1 wt% (Fig. 4), possibly being part of the LDH structure by replacing Mg^2+^.^33^

Al and Si exhibit only a slightly higher content in the BFS particle in comparison to the gel phase, while the Na content is near-zero in the BFS and slightly higher in the gel phase. Ca, Na, and Si are more evenly distributed but Ca is evidently present at lower concentration in the hydrated rims around the slag particles. The CaO–SiO_2_–Al_2_O_3_ ternary diagram (Fig. 3) of the binder gel indicates mainly a C–(A)–S–H composition. The average Ca/Si ratio of the binder is 2.0, which is at the high-end of typical Ca/Si ratios of alkali activated BFS binders,^34^ but is consistent with the presence of Ca(OH)_2_ in the activator blend. At the same time, the Ca content in the binder has high variation and does not cluster to a certain value.

The bright particulates visible in Fig. 2B contain elevated concentration of Fe as shown in Fig. 4. It is noteworthy that all the Fe-rich particles are present within the binder gel, but not inside the BFS particles. Furthermore, there appears to be no systematic overlapping with Fe-rich regions and other elements (Fig. 4), which indicates that Fe could be present as evenly distributed small entrained metallic droplets (nano- and micron-sized particulates). The overall Fe content in the binder gel is ∼2 wt% The speciation of the Fe in the sample is discussed more in detail in the XPS section.

**Fig. 5:**
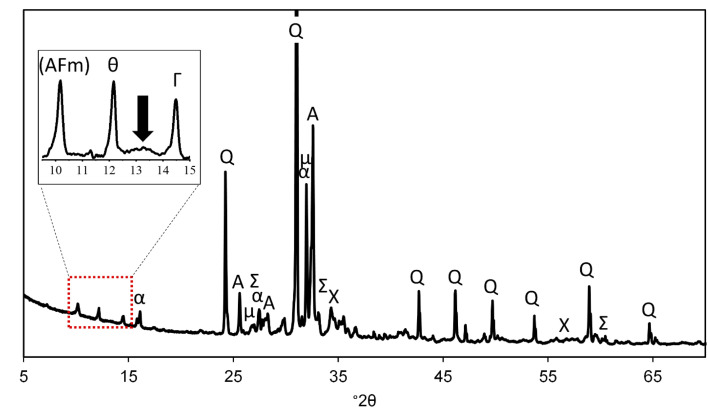
Diffractogram of roof tile sample. Q = quartz (SiO_2_), Σ = thenardite (Na_2_SO_4_), Χ = calcite (CaCO_3_), α = anorthoclase ((Na, K)AlSi_3_O_8_), A = albite (NaAlSi_3_O_8_), μ = microcline (KAlSi_3_O_8_), θ = 3CaO·Al_2_O_3_·0.17CaSO_4_·0.17Ca(OH)_2_·0.66CaCO_3_·*x*H_2_O, Γ = (Mg, Fe, Al)_6_(Si, Al)_4_O_10_(OH)_8_. Peak marked with “(AFm)” did not fit any specific AFm phase but would be likely in this angle range. The arrow in the inset shows the position of the main peak of various Mg–Al LDH phases.

The diffractogram of the roof tile sample is shown in Fig. 5. Most of the identified phases (quartz (#04-014-7569), anorthoclase (#00-009-0478), albite (01-089-6423), and microcline (#01-076-0918)) are related to the aggregates used for the preparation of mortar. Calcite (#01-085-1108) and thenardite (#00-0005-0631), on the other hand, represent efflorescence or subflorescence salts due to carbonation and weathering; similar products have been detected from outdoor building materials in Sweden.^35^ Thenardite is water-soluble anhydrous sodium sulfate, which is possibly a sink of sulfur in the studied sample. It may inflict damage to construction material due to volume changes and pressure caused by phase change, hydration, or crystallization.^36^ However, no such damage could be detected from the studied roof tile specimen and no visible efflorescence was present on the sample surface when sampling was conducted (see Fig. 1). The identification of signals at 10–20° 2*θ* range is less clear: there is an indication of presence of AFm-type and clinochlore phases, but the identification was based on single main peak only (inset in Fig. 5). Also, a weak and broad signal of various Mg–Al LDH phases, such as quintinite, is observable at ∼13° 2*θ* indicating that those phases have poor long-range order.

Upon carbonation, the main reaction product is calcium carbonate with three possible crystalline polymorphs: calcite, aragonite and vaterite, whose formation depends on the concrete pH, temperature and supersaturation, for instance,^37^ and the presence of impurities or admixtures.^38–40^ Of the polymorphs, calcite is the most stable, and all the calcium carbonate present in studied sample was calcite. Also, the formation of sodium carbonates is possible upon carbonation, but in the analyzed sample they were absent.

### Chemical structure

**Fig. 6:**
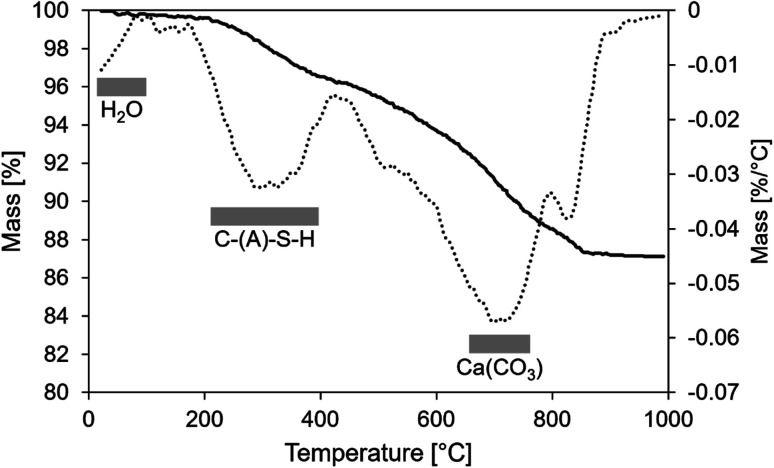
TGA (solid lines, left axis) and DTG (dashed lines, right axis) curves of aged roof tile sample.

The TGA and DTG of the mortar are shown in Fig. 6. Overall, the weight loss up to the temperature of 992 °C was minor: approximately 4%. The weight loss below 100 °C was less than 0.3 weight%, indicating a low amount of capillary pore water and phases with bound water. The peaks between 200–400 °C and 500–800 °C are likely related to the decomposition of C–(A)–S–H gel and calcium carbonate phases, respectively.^41^ DTG peaks related to decomposition of LDH phases appear around 200 and 400 °C and could be related to the presence of hydrotalcite or quintinite. However, the major contribution in this temperature range is likely related to C–(A)–S–H gel. Ben Haha *et al.*^42^ observed that the peaks of LDH phases in TGA were much less distinct if BFS contained less MgO. The BFS used here contained a relatively high amount of MgO (∼9.3 wt%),^43^ which could lead to the Mg–Al LDH formation, but their structure is likely disordered.

**Fig. 7:**
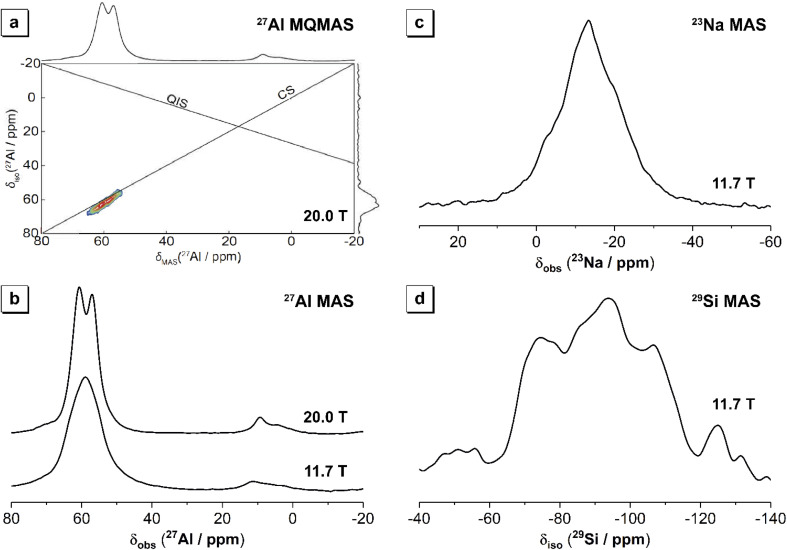
(a) ^27^Al MQMAS NMR data (*B*_0_ = 20.0 T, *ν*_R_ = 20 kHz) with the isotropic projection shown on the vertical axis and the ^27^Al MQMAS NMR data acquired at *B*_0_ = 20.0 T shown on the horizontal axis, (b) ^27^Al MAS NMR data (*B*_0_ = 20.0 T, *ν*_R_ = 20 kHz, and *B*_0_ = 11.7 T, *ν*_R_ = 12.5 kHz, as marked), (c) ^23^Na MAS NMR data (*B*_0_ = 11.7 T, *ν*_R_ = 12.5 kHz), and (d) ^29^Si MAS NMR data (*B*_0_ = 11.7 T, *ν*_R_ = 12.5 kHz) for the aged roof tile sample.

The ^27^Al MAS NMR data (Fig. 7) acquired at high field (20.0 T) for the aged roof tile sample show two distinct but overlapping resonances centred at *δ*_obs_ = 60.8 ppm and 57.1 ppm, respectively, arising from Al sites in tetrahedral coordination within an aluminium-substituted calcium silicate hydrate (C–A–S–H) gel. The observation of multiple tetrahedral Al resonances in the ^27^Al MAS NMR data acquired at high field (20.0 T) is confirmed by the extensive quadrupolar broadening occurring in the data obtained at *B*_0_ = 9.4 T, where only significantly ordered sites will exhibit a quadrupolar lineshape.^44^ It is also consistent with previous observations of multiple Al^IV^ sites in C–A–S–H gels within alkali-activated slag.^45^ The resonances at *δ*_obs_ = 60.8 ppm and 57.1 ppm are attributed to Al in bridging tetrahedra (q^2^) and Al in cross-linked bridging tetrahedra (q^3^) within the aluminosilicate chains in the C–(N)–A–S–H gels, respectively.^46–48^

A small, low intensity shoulder on these overlapping resonances is also observed at *δ* = 70.0 ppm, which may result from Al in a q^2^ site distinct from that observed at *δ*_obs_ = 60.8 ppm, arising due to differences in the local coordination spheres of q^2^ bridging sites due to clustering of different charge-balancing cations, as seen previously for alkali-activated slag cements aged for 2 years.^45^

Resonances arising from Al in octahedral coordination are observed at *δ*_obs_ = 9.3 ppm and 4.4 ppm, the former of which can be assigned to Al in the LDH phases.^46,49^ The broadness of the resonance at *δ* = 9.3 ppm indicates that the LDH phases have low order and no single LDH phase has crystallized by time, which is in accordance with XRD analysis. The resonance observed at *δ*_obs_ = 4.4 ppm is attributed to Al in the “third aluminate hydrate” (TAH), thought to be an amorphous nanoscale aluminate hydrate phase precipitated at the surface of the C–(N)–A–S–H gels within alkali-activated slags and other calcium-rich binders,^50^ and/or Al in silicate-bridging [AlO_2_(OH)_4_]^5−^ sites in C–A–S–H.^51^

^27^Al MQMAS NMR data (Fig. 7) shows that the overlapping tetrahedral Al resonances are primarily broadened along the chemical shift (CS) axis, with minimal broadening along the quadrupolar induced shift axis. This indicates that the Al sites differ primarily to differences in chemical shift (related to chemical connectivity) rather than quadrupolar broadening (related to symmetry), corroborating the assignment of these resonances to Al in bridging tetrahedra (q^2^) and Al in cross-linked bridging tetrahedra (q^3^) within the aluminosilicate chains in the C–(N)–A–S–H gels.^46–48^

The ^29^Si MAS NMR data for the aged roof tile sample (Fig. 7) shows a broad resonance spanning from *δ*_iso_ = −60 ppm to −120 ppm, attributed to a distribution of Q^4^(*m*Al) environments. The broad resonance can be attributed to overlapping resonances due to Q^2^, Q^2^(1Al), Q^3^ and Q^3^(1Al) sites within a sodium and aluminium-substituted calcium silicate hydrate (C–(N)–A–S–H) gel,^52,53^ consistent with previous observations for Portland cement blended with blast furnace slag, and sodium silicate-activated slag cements.

The ^23^Na MAS NMR data for the aged roof tile sample (Fig. 7) shows a broad resonance spanning from *δ*_iso_ = 0 ppm to −30 ppm, and centred at *δ*_iso_ = −13 ppm. This broad resonance is attributed to overlapping resonances, one of which is due to Na^+^ ions within a (C–(N)–A–S–H) type gel, charge balancing, tetrahedrally coordinated Al^3+^ ions situated in fully polymerised Q^4^ Si sites.

The Fe speciation in old cement samples is of interest as it can act as an inert or reactive binder-forming component depending on its original speciation, but also on the prevalent conditions during the concrete life-time.^22,54^ BFS typically contains iron, which is mainly present as metallic (Fe^0^) nano- to micrometer-sized particles.^22,54^ It is speculated that corrosion behavior of Fe^0^ particles in BFS cements could be similar to that of iron and steel reinforcement, thus giving information about the long-term resistance of reinforcements in BFS concretes also. Previous study showed that no Fe^0^ oxidation was observed for alkaline activated BFS hydrated at laboratory conditions for 28 days.^54^ Similarly, only little Fe^0^ oxidation was observed for blended Portland cement and BFS (36–65 wt%) concrete which had been exposed to river water for 7 years.^22^ However, complete oxidation of Fe^0^ was observed for blended Portland cement and BFS (∼71 wt%) concrete which had been exposed to sea water for 34 years.^22^ The differences were explained by the presence of chloride in sea water which can accelerate the Fe^0^ oxidation and form *e.g.*, Fe-hydroxides and Fe–Si-hydrogarnet.

**Fig. 8:**
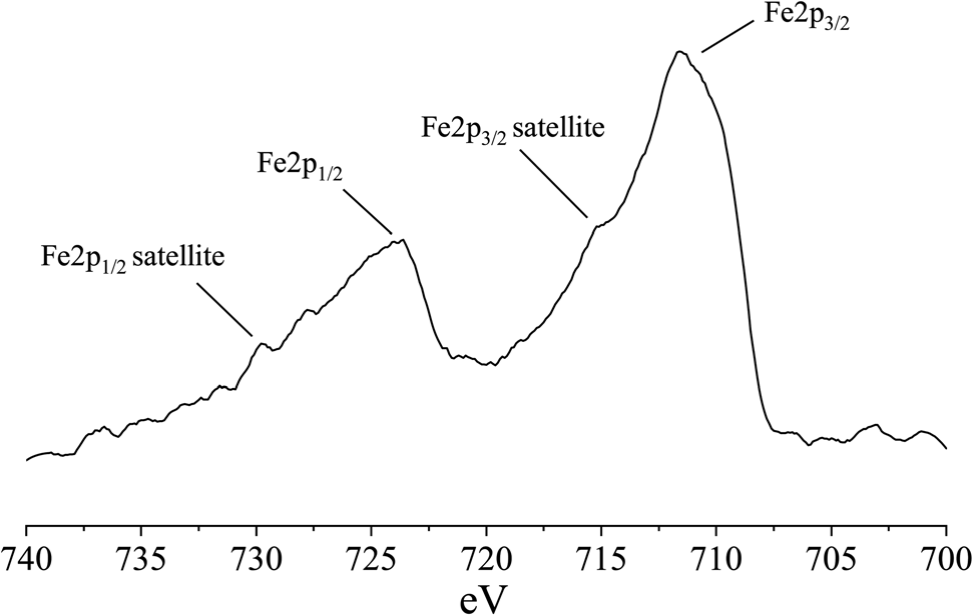
Fe 2p XPS spectrum of the roof tile sample.

In our sample, the iron content is 2.6 wt% in terms of Fe_2_O_3_ (Table 1), which is substantially high considering that the XRF analysis result also includes the aggregates in the roof tile sample. The roof tile has been exposed only to rainwater and snowmelt, which are assumed to contain negligible concentration of chloride, thus major oxidation should not be expected based on the above-mentioned studies. However, based on XPS analysis (Fig. 8), Fe is present as oxidized Fe(ii) and Fe(iii). The Fe 2p spectrum shows two main signals: Fe 2p_3/2_ at ∼711 eV and Fe 2p_1/2_ at ∼724 eV, and two transfer satellite signals at 715 eV and 729 eV.^55,56^ The satellite at 715 eV indicates that iron is mainly present as Fe(ii), although some Fe(iii) is also present, as the Fe 2p_3/2_ signal at ∼711 eV is broad and thus likely contains signals for both Fe(ii) and Fe(iii).^55,56^ Furthermore, the background around 720 eV is clearly higher than at 705–700 eV indicating that there is a Fe 2p_3/2_ satellite signal of Fe(iii) in that range.^55,56^ Fe^0^ should have a signal between 706–707 eV,^55^ but no signal is detected in that range, showing that it is not present in the sample. However, as the XPS analysis measures only the first 10 nm of the surface of the powdered roof tile particles, any Fe^0^-rich particles located deeper inside the sample particles could nevertheless possess most of the mass of Fe detected by the XRF analysis.

**Fig. 9:**
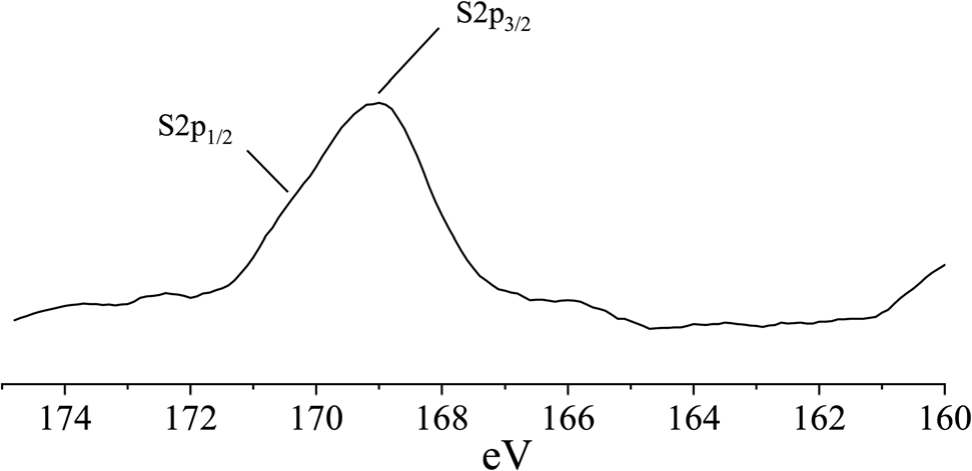
S 2p XPS spectrum of the roof tile sample.

Sulfur is usually present in reduced (*i.e.*, sulfidic) form in slag due to the reducing conditions of slag production. Fig. 9 shows a S 2p_3/2_ signal at ∼169 eV, which indicates the presence of oxidized S(vi) species in metal sulfates.^55^ There is also a minor shoulder peak at 170 eV, which can be assigned to the S 2p_1/2_ signal of S(vi) in metal sulfates.^57^ The sulfate can be associated to Na_2_SO_4_, in agreement with the XRD analysis (Fig. 5).

### Carbonation

**Fig. 10:**
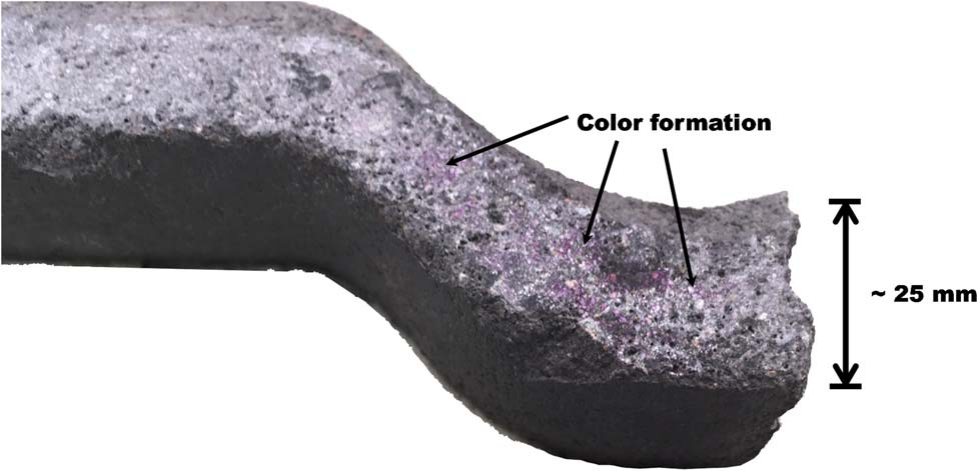
A cross-section of the roof tile in the carbonation depth determination: pink color development (*i.e.*, pH ≈ 8–10) took place in the inner pores of the tile.

The phenolphthalein test exhibited a weak pink color development locally in the pores, whereas the bulk phase of the binder remained colorless (Fig. 10). Thus, the inner parts of the mortar have remained in the pH range higher than 8 (*i.e.*, not carbonated despite the detection of CaCO_3_ by the other analyses). This result indicates a remarkable carbonation resistance ability as the studied structure is only approximately 25 mm thick and it has been exposed to an aggressive outdoor environment for three decades. Refined capillary pores and low permeability are the key aspects of durability of cementitious binders. Here, an extrusion pressure process was used during the manufacturing of the tile, which could provide a relatively dense matrix due to lower water/binder ratios, and thus increase durability compared to cast mortars and concretes. However, 3D printing of mortars by extrusion (although modern 3D printing of mortars is not comparable to the method used for tile manufacturing) is known to cause more defects in comparison to conventional casting, and thus it is difficult to assign if the observed good durability is intrinsic to the manufacturing process.

The Mg–Al LDH phases have been observed to act as an internal CO_2_ and chloride sorbents in AAMs, which can improve AAM resistance to carbonation under natural ambient conditions.^43,58^ The high MgO content of the BFS used here can increase the Mg–Al LDH formation and lower the porosity of the binder,^42^ and thus improve the durability.

Based on the visual observation, the freeze–thaw durability has obviously been excellent as samples contained no cracks or deterioration matrix. This is also likely due to the low permeability.

## Conclusions

The phase assemblage of a 30 year-old alkali-activated BFS mortar was characterized. The results show that the binder consists of C–A–S–H gel with average Ca/Si ratio of 2.0. The ^29^Si MAS NMR data showed a broad resonance attributed to Q^2^, Q^2^(1Al), Q^3^, Q^3^(1Al) and Q^4^ sites, demonstrating that Si is present in numerous coordination states in the C–A–S–H gel and has not been transformed into a single phase over time. Also, no zeolites or N–A–S–H gel were detected. As a secondary reaction product, Mg–Al LDH with Mg/Al ratio of 2 and limited long-range order structure was detected at the boundaries of reacted BFS particles. Mn was associated with the Mg–Al LDH regions, indicating that Mn from the slag has partially replaced Mg in the LDH structure. Calcite was detected, indicating carbonation to some extent, however the inner parts of the sample showed remarkable resistance to carbonation after an extended time in service. This is expected to be partly due to the dense microstructure of the sample. The results indicated that while iron has been originally present as small Fe^0^ particulates, it has oxidized into Fe^2+/3+^ during the cement lifetime and is evenly distributed in the binder gel showing that iron can be a reactive component in AAMs rather than inert as it is often considered.

## Conflicts of interest

There are no conflicts to declare.

## Supplementary Material
